# Situating Sub-Saharan Africa Within Intra-Operative Innovations in Neurooncology

**DOI:** 10.3389/fsurg.2022.889965

**Published:** 2022-06-23

**Authors:** James A. Balogun

**Affiliations:** Division of Neurological Surgery, Department of Surgery, College of Medicine, University of Ibadan and Department of Neurological Surgery, University College Hospital, Ibadan, Nigeria

**Keywords:** neurooncology, intra-operative ultrasound, brain mapping, 5-ala, Sub-Saharan Africa, 5-ALA = 5-aminolevulinic acid

Surgical neurooncology has witnessed impactful innovations and refinements of previous techniques and discoveries over the last few decades. The pre-operative evaluation of patients has evolved to include the physiologic and functional imaging of the brain (1). This is in addition to the traditional anatomical brain imaging, permitting more robust surgical planning, such that brain tumors are not only better localized and characterized, but the functions of the surrounding normal brain are also elucidated. The intra-operative armamentarium at the disposal of the surgical neurooncologist has also widened to include neuronavigation, which has become an invaluable tool, can facilitate pre-operative fibre tracking, and be optimized/synchronized with other Intra-operative imaging modalities such as CT/MRI/ intra-operative ultrasound (IOUS). The limited availability of neuronavigation in Africa (2), SSA inclusive, makes familiarity with neuro-anatomy inevitable, ensuring the use of craniometric reference points for surgical planning, with access to soft wares such as Osirix/Horos an additional boost (3, 4). Other innovations include microscopes of varying sophisticated functionalities, tractography, brain mapping, Fluorescence guided tumor resections, robotic assistance for tumour biopsy, and laser interstitial thermal therapy (LITT). All of these innovations have been demonstrated to empirically improve surgical safety and increase the extent of tumor resections (5, 6), while preserving the integrity of the surrounding normal brain. The post-operative care of brain tumor patients is essentially anchored on the provision of adjuvant therapy, usually through a multidisciplinary brain tumor board.

The Sub-Saharan Africa (SSA) continent, by 2019 World Bank Data is estimated to have a population of 1.107 billion with a projected population doubling by 2050. The population is very diverse with heterogeneous ethnic and religious compositions. The health allocation by countries varies from 6%–15% of the national budget, necessitating that payment for healthcare is basically “out of pocket”. The increasing burden from brain tumors has continued to be demonstrated through the institutional, country and regional data of brain tumors, demonstrating similar tumor spectrum as other regions (7) and also validated with modeled statistics, though this reporting is limited due to the paucity of tumor registries (8, 9). The challenges encountered in the care of patients with brain tumors have been identified in varying reports and there are concerted collaborative efforts to overcoming them including the formation of the Society for Neurooncology Sub-Saharan Africa (SNOSSA) (10).

The focus of this piece is to situate the SSA within some of the innovations that have impacted on the intra-operative tools available in neuroncology, identify the challenges of access and use in SSA and proffer ways by which these developments can be utilized to impart on surgical neurooncology, and ultimately influence the outcome of brain tumor patients in SSA.

Intra-operative brain mapping either under general or awake anaesthesia, initially employed in epilepsy surgery, has revolutionized the surgery of brain tumors located within and around the eloquent regions of the brain. The direct electrostimulation (DES) of different brain functions under an awake setting, has in particular, been arguably described as the contemporary gold standard in glioma surgery, which optimizes the delicate onco-functional balance (11, 12). It has been used to define motor, language and sensory functions and also extended to testing neuro-cognitive functions (13, 14). The efficacy and utility of the technique has been demonstrated across all age groups including children (15, 16). The ability to map brain functions has resulted in improved extent of resection of tumors as well as preservation of functions along tumor location thereby improving the quality of life of patients (12, 17). There is a spectrum of these devices deployed in intra-operative monitoring which vary from the use of battery/electric powered, stand alone cortical/subcortical stimulators to the full multi-parametric electrophysiology machines with the added advantage for corticography. The availability of these devices in SSA appear to be non-existent as there have not been documentation of the deployment of this technique, which may be due to the high cost of purchase of the cortical stimulators and consumables such as the probes and perhaps the unavailability of a lease system that helps the mitigates the challenge of outright purchase, such as utilized in South Africa in the provision of intraoperative neurophysiologic monitoring for brainstem spine surgeries (18). Thus, the established advantages of brain mapping have not been fully appreciated in SSA countries, although there have been reports of awake craniotomy without brain mapping, with positive impacts on the elimination of the need for general anesthesia and reduction of both operative and overall hospital cost (19, 20). Thus, the exposure of surgeons to this technique, in-country/regional availability of lease of these devices, availability of neurophysiologists and multi institutional and industry collaboration will be the keys to unlocking the adaptation and utility of these techniques in SSA.

There has been extensive debate on the utility of intra-operative imaging tools such as the CT scan and MRI of varying magnet capacity, especially the latter, which though provides a great intra-operative guide to extent of resection but is limited in its availability due to the high cost of installation and maintenance, the need for special instruments and extra support staff (6). There is therefore an understandable increasing advocacy for the use of IOUS, because of its ready availability and relatively cheaper cost (21). Though the issues of user-dependency, a somewhat steep learning curve, anatomic orientation and scan quality have been noted with it's use (22), the advantages of real time intra-operative evaluation has continue to make it one of the neurosurgeons' delight in the modern operating room (23). These include the removal of the effect of brain shift with unpredictable distortions after craniotomy and tissue removal noted with neuronavigation, the ease of use with minimal operational flow interruptions, and a lack of radiation exposure. Further improvement in technology has permitted the fusion of ultrasound and neuronavigation images, allowing for navigated ultrasound pictures (24), as well as contrast enhanced ultrasound images with clear elucidation of the blood vessels. Thus, IOUS appears to be a possible “equalizer” in the surgical care of patients in the HICs and LMICs. There is however very little in the literature to validate the ready availability of IOUS across SSA. This may be due to the identified challenges with IOUS use, especially the lack of familiarity with the orientation of the images, which differs from the axial, coronal and sagittal view orientations that neurosurgeons are acquainted with. This situation can be addressed by organized training sessions and mentored support (physical or virtual) during the initial period of use in neurosurgeon's practice. Indeed, SSA must strive to be at the forefront of future innovations around the IOUS.

Fluorescence guided resections have progressively become an important adjunct in the surgery of high-grade gliomas and there have been explorative adaptations of its use in low-grade glioma, brain metastases and pediatric brain tumor surgeries (25–27). This involves either the use of the 5-Aminolevulinic acid (5-ALA), administered orally, pre-operatively, or the Fluorescein, which is administered intravenously, intra-operatively. Both fluoresceins have been proven to improve the extent of tumor resection and thus improve the overall survival of patients. A combination of both the 5-ALA and Fluorescein for the synergistic effect of achieving improved visualization of both the tumor and the surrounding brain has also been utilized (28). The limitation of fluorescein-guided resections is the expensive cost of the microscope blue light filters and to an extent, the prevailing patency on the most commercially available tablet. While there are efforts to use the yellow light of the microscope for visualization of the fluorescing cells, however, more exciting is the reports of the use of headlamp/loupes for fluorescein guided resections (29–31), a situation that is likely to make fluorescein guided surgery available in most LMICs including SSA. The New York group also tested the efficacy of the use of the common blue light flashlight in identifying fluorescein cells (29), and reported it did identify the malignant cells to some degree. However, this will require further exploitation and validation and will certainly provide a cheaper alternative, even to the loupes and thus, make the use of fluorescein more available.

It is therefore pertinent to remain optimistic and be forwarding looking as surgical neurooncologists in SSA despite the myriad of challenges we are faced with. The practice of reasonable contemporary surgical neurooncology does not look as distant as it appears. We must continue to strive for excellence and position ourselves to be in the center of the evolving innovations with emphasis on the peculiarities of our environment.

## Data Availability

The original contributions presented in the study are included in the article/Supplementary Material, further inquiries can be directed to the corresponding author/s.
